# Telomerase activity as an adjunct to high-risk human papillomavirus types 16 and 18 and cytology screening in cervical cancer

**DOI:** 10.1038/sj.bjc.6603375

**Published:** 2006-10-24

**Authors:** U Kailash, C C Soundararajan, R Lakshmy, R Arora, S Vivekanandhan, B C Das

**Affiliations:** 1 Division of Molecular Oncology, Institute of Cytology and Preventive Oncology (ICMR), Sector 39, NOIDA – 201301, UP, India; 2 Department of Neurobiochemistry, All India Institute of Medical Sciences, New Delhi 110029, India; 3 Department of Cardiac Biochemistry, All India Institute of Medical Sciences, New Delhi 110029, India; 4 Department of Obstetrics and Gynecology, Lok Nayak Hospital, Maulana Azad Medical College Campus, Bahadur Shah Zafar Marg, New Delhi 110002, India

**Keywords:** telomerase activity, human papillomavirus, cervical cancer, screening, cytology

## Abstract

Telomerase is a ribonucleoprotein comprising an RNA template, the telomerase-associated protein and its catalytic subunit, human telomerase reverse transcriptase (hTERT). Telomerase activation is a critical step in cellular immortalisation and development of cancer. Enhanced telomerase activity has been demonstrated in cervical cancer. In the present study telomerase activity and hTERT mRNA expression were evaluated and correlated with the presence of human papillomavirus (HPV) infection and cytological changes in the cervical lesions. Telomerase activity was assayed by telomeric repeat amplification protocol, hTERT mRNA expression by reverse transcriptase polymerase chain reaction and presence of high risk HPV (HR-HPV) infection by polymerase chain reaction. Out of 154 cervical samples of different cytology, 90 (58.44%) were positive for HR-HPV types 16/18, while among 55 normal cervical scrapes, 10 (18.18%) were HPV DNA positive. All 59 invasive cancer samples showed a very high telomerase activity. Among dysplasia, seven (63.6%) mild dysplasia, 18 (100%) of moderate, 20 (100%) of severe dysplasia and 6 (100%) carcinoma *in situ* (CIS) samples were positive with mild to moderate to high to very high telomerase activity respectively. Seven (12.7%) samples of apparently normal cervical scrapes were weakly positive for telomerase activity. We observed a good correlation (*P*<0.001) between telomerase activity and HR-HPV 16/18 positivity with a sensitivity of 88.1% for HPV and 100% for telomerase activity. It is suggested that telomerase activity may be used as an adjunct to cytology and HPV DNA testing in triaging women with cervical lesions.

Cervical cancer is one of the most common causes of mortality among women worldwide with an annual incidence rate of approximately 400 000 cases and 200 000 deaths per year (Parkin *et al*, 2001). The greatest burden of the disease is in developing countries where lack of organised screening facility contributes to nearly one quarter of all female cancers. In developed countries Pap smear screening is associated with a 75% reduction in the incidence of cervical cancer (Kurman *et al*, 1994).

Clinical and molecular epidemiological studies demonstrated convincingly that certain types of human papillomaviruses (HPVs) are the primary causal agents in the development of cervical carcinoma (Bosch and de Sanjose, 2002; Munoz *et al*, 2003). So far, more than 100 different types of HPV have been found and their nucleotide sequences have been characterised. Among them, 30 types have been linked to the development of anogenital cancers. Human papillomavirus types have been classified into ‘low’ and ‘high’ risk types based on their potential to induce tumourigenic transformation. HPV types 16,18,31,33,35,39,45,51,52,56,58,59,68,73 and 82 are considered as high risk types while 6,11,40,42,43,44,54,61,70,72,81 and CP6 108 as low risk types (Munoz *et al*, 2003). Among high risk HPV types, HPV 16 and 18 are considered to be carcinogenic agents and are strongly implicated with the development of cervical cancer (IARC Monographs Working Group, 1995; zur Hausen, 2001).

Studies on the oncogenic potential of these HPV types have clearly demonstrated that HR-HPVs are a necessary cause for the development of cervical cancer (Schiffman *et al*, 1993; Bosch *et al*, 1995) but only a small proportion of women with cytological abnormalities or infected with HR-HPV types will eventually progress to invasive cancer with majority of infections is cleared spontaneously, which suggests the view that although infection with HPV is essential but it may not be sufficient for the development of cervical cancer. Only persistent HPV infection may lead to the development of invasive cervical cancer. Screening of cervical cancer is essentially carried out by the Pap smear test, which although effective in detecting high risk premalignant and malignant cervical cells, it suffers from high false negative rates, intra- and interpersonnel and laboratory variations. New screening strategies, including testing for HR-HPV DNA as an adjunct to cytology to triage and monitor cervical lesions have been advocated. Involvement of other cellular and molecular events in the HPV-induced cervical carcinogenesis turned the focus to identify other molecular markers for effective screening and diagnosing cervical pre and invasive malignancies.

Recent observations support the concept that activation of telomerase is a critical step in cellular immortalisation and cancer. Telomeres, the ends of chromosomes are specialised DNA-protein complexes, composed of TTAGGG repeats. They play an important role in genomic integrity and stability by preventing the recognition of chromosomal ends as double-stranded DNA breaks. Telomerase synthesises (TTAGGG)_n_ DNA repeats onto chromosome ends. It is a ribonucleoprotein complex, consisting of an internal RNA component human telomerase RNA (hTR), a catalytic protein subunit, human telomerase reverse transcriptase (hTERT) and other associated proteins namely hTP1 etc. Most adult somatic cells exhibit low or no telomerase activity and thus experience progressive telomere attrition with each round of cell cycle. In contrast, germline cells, differentiating cells and tumour cells have elevated or detectable levels of telomerase activity and have long or stable telomeres. Consequently, telomerase may be required for the long-term proliferation of tumours. Stabilisation of telomere lengths by activation of telomerase is thought to be the key mechanism of indefinite cell proliferation and immortalisation. Telomerase activity has been reported in different tumour types (Kim *et al*, 1995). Elevated levels of telomerase activity have been detected in invasive cervical carcinomas, as well as in high-grade cervical dysplasias. A few investigators also reported low telomerase activity in normal, inflammatory and premalignant cervical lesions (Zheng *et al*, 1997; Wisman *et al*, 2000).

The main transforming genes of HPV 16 and other HR-HPV types are E6 and E7. HPV E6 protein generally target and degrade tumour-suppressor protein, p53, through ubiquitin pathway (Scheffner *et al*, 1990, 1993; Werness *et al*, 1990). However, it has also been reported to bind to a number of other cellular proteins and has functions that are independent of p53 degradation including the activation of telomerase (Rapp and Chen, 1998). Sprague *et al* (2002) indicated that HPV-16 by itself does not necessarily cause telomerase activation in cervical keratinocytes, but rather, supports a model in which HPV-16 facilitates telomerase activation in conjunction with other viral or cellular changes over time. Given that HPV infection has been associated with majority of the cases of invasive cervical cancers, it may be deduced that, telomerase activation may be a critical pathway by which HPV infection facilitates malignant transformation of the cervical epithelium, making it an ideal marker for cervical cancer screening. In India, cancer of the uterine cervix is the major cancer in women and infection of HPV has been detected in more than 98% cases (Das *et al*, 1992a, 1992b). There are, however, only a few studies from India where an attempt has been made to augment skeletal cervical cytology screening programme. Recently, Arora *et al* (2005) suggested that HR-HPV detection can be utilised as an adjunct to routine cytology screening programmes to identify ‘high risk’ women who have concurrently negative Pap smears but may harbour oncogenic HPV infection and/or are more likely to develop cervical intraepithelial neoplastic lesions.

In the present study, we investigated whether the status of HPV infection, telomerase activity, hTERT, hTR and hTP1 mRNA expressions in cervical tissues and/or cervical scrapes have clinical value in the triage of women or useful as an adjunct to cytology, particularly undefined or atypical squamous cells of undetermined significance (ASC-US) and mild, moderate or severe dyskaryosis.

## MATERIALS AND METHODS

This study was conducted in 154 tissue biopsies from patients in the age group 25–60 years and included 59 invasive cervical carcinomas, 55 dysplastic tissues belonging to various grades namely, mild (*n*=11), moderate (*n*=18), severe dysplasias (*n*=20) and CIS (*n*=6) cases and 40 normal cervical tissue controls. The study also included 55 cervical scrapes from asymptomatic, normal healthy women. All the biopsies were colposcopy-directed, and were either histopathologically or cytologically confirmed by the pathologists. Before taking punch biopsy, cervical scrapes were taken from all invasive cancer patients.

The biopsy specimens of cervical carcinomas were collected from women attending the ‘cancer clinic’ at Obstetrics and Gynaecology out-patient department (OPD) of Lok Nayak Hospital, New Delhi. The clinical staging of the tumours ranged from stage I to stage IV and their histological tumour grading was either of poor, moderate or well-differentiated squamous cell carcinomas and adenocarcinomas and subtypes thereof. Control biopsy specimens were collected from asymptomatic, apparently normal women visiting OPD and undergoing hysterectomy for gynaecological reasons other than cervical cancer such as fibroids, dysfunctional uterine bleeding etc. Cervical scrapes were also collected from asymptomatic normal women coming for checkups other than gynaecological complaints. Informed consent was obtained from each patient before recruitment for the study.

Biopsies and the scraped cervical cells were collected in chilled PBS and transported from cancer clinic to the laboratory on ice. Each biopsy specimen was bisected after grossing by pathologists; one-half was subjected to histopathological examination after fixing the sample in 10% formaldehyde. The remaining half was employed for DNA and RNA extraction. The whole procedure was completed within 3 h from the time of collection.

### HPV analysis

Genomic DNA was extracted from the cervical tissues/scrapes using Proteinase K/phenol-chloroform method. Human papillomavirus detection was carried out by polymerase chain reaction (PCR), using the consensus primers MY09 and MY11, with an expected product size of about 450 base pairs (bp), for the amplification of the most conserved LI region of the HPV genome. A gene fragment of the *β*-globin was used as an internal control to check the integrity of the specimens. Further HPV typing was carried out using type-specific primers for HPV types 16, 18, 6 and 11 (Das *et al*, 1992a). Polymerase chain reaction products were separated electrophoretically on an ethidium bromide stained 3% Nusieve agarose gel. Each PCR reaction included a positive and a negative control.

### Estimation of telomerase activity by TRAP assay

A portion of the tissue grinded in liquid nitrogen (40–100 mg) was suspended in 200 *μ*l ice-cold CHAPS lysis buffer (10 mM Tris-HCl pH 7.5, 1 mM MgCl_2_, 0.1 mM phenylmethylsulphonyl fluoride, 1 mM EGTA, 5 mM *β*-mercaptoethanol, 0.5% CHAPS and 10% glycerol). After 30 min of incubation on ice, lysates were centrifuged at 10 000 r.p.m. for 20 min at 4°C, the supernatant was aliquoted and stored at −70°C until used for assay.

Telomeric repeat amplification protocol (TRAP) assay was carried out as described by Kim *et al* (1995) with minor modifications in 50 *μ*l reaction mixture containing (20 mM Tris-HCl (pH 8.3), 1.5 mM MgCl_2_, 63 mM KCl, 0.005% Tween-20, 1 mM EGTA, 50 *μ*M each of dNTPs, 1 *μ*g of T_4_ gene 32 protein (Boehringer Mannheim, Mannheim, Germany), bovine serum albumin (1 mg ml^−1^), 0.1 *μ*g of TS primer (5′-AATCCGTCGAGCAGAGTT-3′) and [*α*-P32]dCTP10Ci/*μ*l (specific activity 3000 Ci mmole^−1^) with 5 *μ*g of protein. After telomerase mediated strand formation 0.1 *μ*g of CX primer was added at 72°C and PCR amplification was performed. Lysate from HeLa cells were used as positive control. Sample treated with RNAse A was treated as negative control. Lysis buffer was used as reagent blank to monitor reagent contamination.

The PCR products were then analysed by electrophoresis on 12% polyacrylamide nondenaturing gel and autoradiographed. The level of telomerase activity was quantified by comparing the density of the ladder signals with the positive control (HeLa cells) and expressed in relative units (RU). Samples which did not give ladder were comparable to negative control and were graded as 0, the ladder which gave stronger signal compared to positive signal as 4+(++++), equal signal as 3+, moderate as 2+ and mild in 1+ RU.

### Expression analysis of telomerase components hTERT, hTR and hTP1

RNA was extracted from the tissue biopsies using TRI reagent according to the manufacturer's specifications. The cDNA was synthesised for each of the sample using 5 *μ*g *μ*l^−1^ of RNA. The reaction was carried out in 20 *μ*l containing 50 ng of random hexamer primers (N6) (Bangalore Genei, Bangalore, India), 250 *μ*mol of each of nucleotides (dATP, dCTP, dGTP and dTTP), 0.1 M of dithiothreitol, 40 U *μ*l^−1^ of RNAse inhibitor (Life Technologies, Carlsbad, CA, USA) and MMLV reverse transcriptase (Gibco BRL, NY, USA). The cDNAs were stored at −70°C until PCR amplification.

Two microlitre of cDNA was taken in a total volume of 50 *μ*l reaction mixture, containing 10 mM dNTPs, 10 *μ*M of each set of primers, 5 U of Taq DNA polymerase, by using gene-specific primers for hTERT, hTR and hTP1 along with the primers for glyceraldehyde-3-phosphate dehydrogenase (G3PDH). The cDNA was amplified for 36, 32 and 30 cycles, respectively, for hTERT, hTR, hTP1 along with house keeping gene G3PDH. The amplified PCR products were resolved by electrophoresis on ethidium bromide stained 1.5% agarose gel and visualised in BIO-RAD gel documentation system.

## RESULTS

### Detection of HPV by PCR

**Figure 1:**
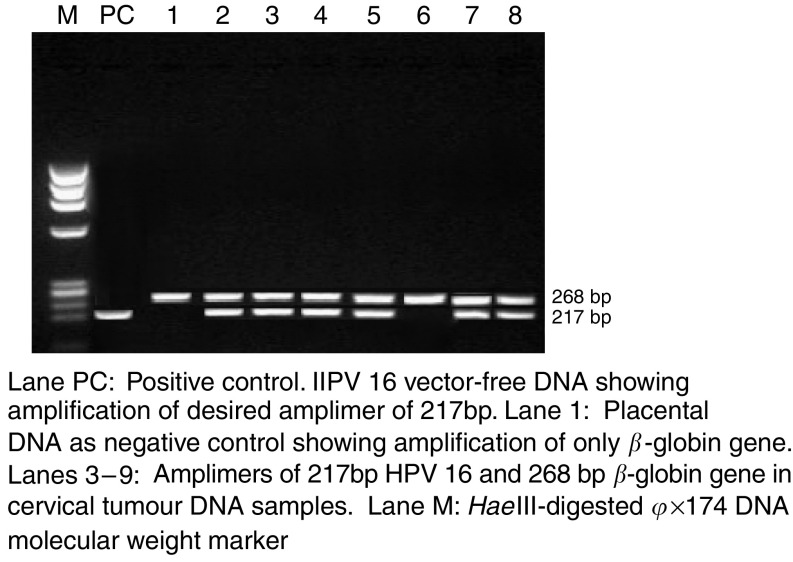
Simultaneous PCR amplification of (1) HPV 16 URR and *β*-globin gene in cervical cancer DNA samples.

In all, 52 out of 59 invasive cervical carcinomas (88.1%), 38 of 55 (69.1%) dysplasias, two (5%) out of 40 normal control tissues and 13 of 55 (23.6%) normal cervical scrapes were positive for HPV by LI consensus primers. Further typing of HR-HPV 16 and 18 was carried out by PCR amplification of most conserved upstream regulatory region (URR) sequence of HPV 16, which results in a PCR product of 217, and 100 bp band HPV 18 E6 region (Figure 1).

Table 1 demonstrates, out of 59 cervical tumour biopsies, 50 (84.7%) were positive for HPV type 16 and two (3.39%) for HPV type 18. None of the invasive tumour samples showed amplification for other two HPV types (6 and 11). Thus, the positivity for HR-HPV types 16/18 as revealed by PCR was 88.14%. Polymerase chain reaction amplification of 55 cervical dysplasia samples revealed 36 cases (65.4%) of HPV type 16. The break-up of HPV type 16 positivity among dysplasia cases was six (54.5%) of mild, 10 (55.5%) of moderate, 20 (76.9%) of severe dysplasia which include five cases of CIS, respectively. two samples (5%) of normal control tissues were positive for oncogenic HPV type 16 and remaining 38 samples were negative for any type of HPV infection as revealed by consensus primers. Polymerase chain reaction amplification for HPV 16 in normal cervical scrapes revealed 10 (18.18%) positive cases out of which one sample (1. 81%) was also positive for HPV 18 (Table 1).

### Telomerase activity

**Figure 2:**
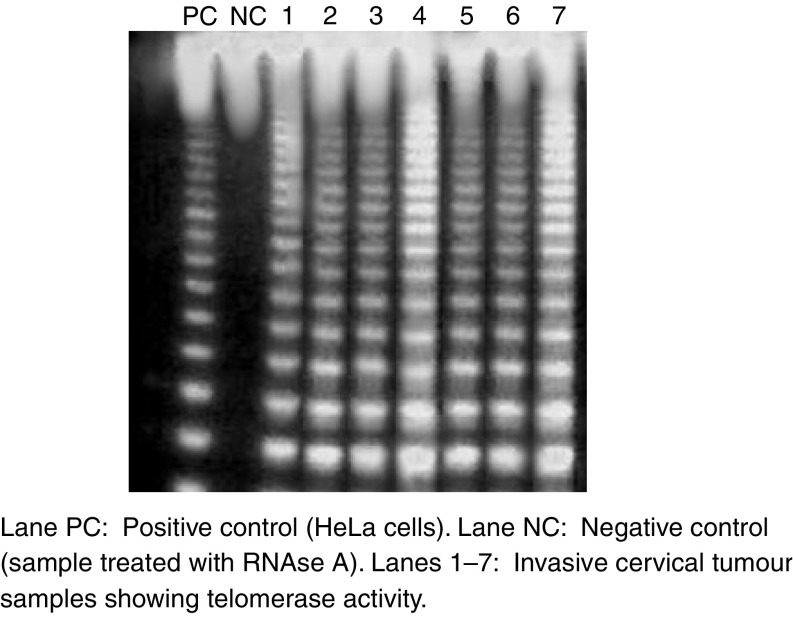
Telomerase activity in cervical tumour tissues. Tissue extracts showing 6 bp ladder from TRAP assay depicting telomerase activity.

All 59 (100%) invasive cervical tumours showed a very high telomerase activity when compared to that of controls (5%). Cervical smears from the same patients also showed similar increased activity. Of the 55 dysplasia samples, seven (63.6%) of 11 mild dysplasia, samples revealed low telomerase activity. While all 18 (100%) moderate, 20 (100%) of severe dysplasia and six (100%) of CIS cases were positive for moderate to high to very high telomerase activity respectively. Out of 40 normal control tissues only two (5%) were positive for telomerase activity but seven cases (12.7%) of apparently normal cervical scrapes showed weak telomerase activity. Telomerase activity in different cervical lesions is shown in Figure 2.

### Telomerase activity and HPV status in cervical lesions

Table 1 demonstrates associations, sensitivity and specificity of telomerase activity and HPV status in normal and abnormal cervical lesions. The HR-HPV 16/18 infection (OR 141.1, *P*<0.001) showed 88.1% sensitivity in invasive carcinomas compared to 100% sensitivity of telomerase activity. It is further observed that as the positivity of oncogenic HPV types 16/18 increased telomerase activity also increased with the increased severity of the disease. Severe dysplasia cases showed 100% sensitivity for telomerase activity compared to 76.9% for HPV. Telomerase activity thus appears to be a better molecular marker than HR-HPV 16/18 in detecting lesions even in mild dysplasia cases. Table 2 shows 88.1% concordance between telomerase activity and HPV status in invasive cervical carcinomas, 69.1% in dysplasias and 100% in normal cervical tissues. The extent of agreement in positivity among ‘normal’ scrape samples of 55 women also showed a good concordance (94.5%) between HPV and telomerase methods.

### Expression analysis of telomerase components hTERT, hTR and hTP1

**Figure 3:**
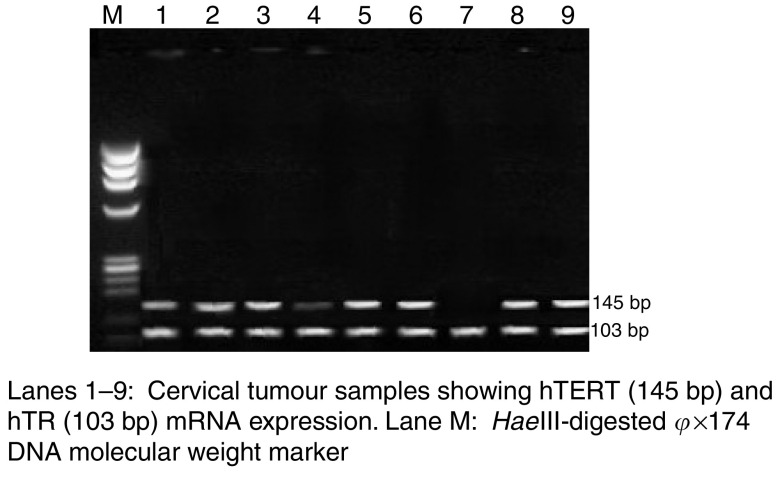
RT–PCR showing hTERT and hTR expression in cervical tumour tissues.

RT–PCR experiments for hTERT, hTR and hTP1 mRNA expressions revealed that both hTR and hTP1 were constitutively expressed in all invasive carcinomas, dysplastic lesions and normal controls of the uterine cervix. However, the expression of hTERT levels was greater in invasive cervical cancer samples and showed an increasing trend with the increasing grade of dysplasia/cervical cancer (Figure 3).

As regards to hTERT, a very high expression levels was observed in all 59 invasive cervical carcinomas, which also well correlated with their high telomerase activity. Human telomerase reverse transcriptase expression in eight mild, 18 moderate, 20 severe dysplastic lesions and six CIS cases ranged from mild to moderate to high and very high levels, respectively. While seven samples of normal cervical scrapes revealed a weak hTERT mRNA expression, none of the normal control tissue samples had any detectable levels of hTERT expression. A specific PCR amplimer of 145 bp for hTERT and a 103 bp for hTR are shown in Figure 3. The extent of hTERT expression shows a significant correlation with the level of telomerase activity and the grade of the disease (*P*<0.001).

### hTERT, hTR and hTP1 mRNA expressions in relation to HPV

When HPV infection in the different categories of cervical lesions was correlated with the expression of telomerase components hTERT, hTR and hTP1 as determined by RT–PCR, a good correlation with the presence of oncogenic HPV type 16 was observed. Human telomerase reverse transcriptase expression was detected in as many as 88 of 90 (97.8%) specimens which were positive for HR-HPV type 16, with the exception of only one (1.11%) of HPV-positive samples not expressing hTERT (*P*<0.001). Human telomerase RNA and hTP1 expressions were found even in HPV negative samples, although not as high as in HPV-positive samples. However, their expression levels increased with increasing severity of the disease.

### Statistics

The Fisher's exact test was used for comparisons between groups. Statistical analysis was carried out using SPSS (version 10) software.

## DISCUSSION

We studied the relationship between HPV status, telomerase activity, hTERT, hTR, hTP1 mRNA expression during the development of cervical cancer through different grades of cervical dysplasia to invasive cervical cancer.

Cytology is considered as the gold standard for the diagnosis of dysplastic and invasive uterine cervical lesions. Pap smear test routinely employed for the diagnosis of premalignant cervical lesions is, however, found to have variable sensitivity and specificity. Testing for oncogenic type of HPVs is another viable method to screen women with cytological abnormalities since HR-HPV infections are considered as the most common causal factor and are reported in more than 90% of cervical cancers (Das *et al*, 1992b; Bosch *et al*, 1995; Bosch and de Sanjose, 2002). We have considered only four HPV types (HPV 16,18,6 and 11) that are highly prevalent in and around Delhi for this study. HPV types 16 and 18 constitute almost 90% of cervical cancers in India and the presence of other high risk types is very low or nil (Das *et al*, 1992a, 1992b; Gopalkrishna *et al*, 2000; Franceschi *et al*, 2005). It has been reported that 10–35% of normal healthy women harbour HPV DNA in their cervical epithelium depending on the screening method, age of the women, number of subjects studied and their geographic locations (Lorincz *et al*, 1986, 1992; Toorn *et al*, 1986). In the present study only 5% of normal control tissues (*n*=40) compared to 18% of (*n*=10) normal cervical scrapes were positive for HR-HPV types 16/18 infection. This observation in the present study is interesting. It is possible that these women who were attending OPD with symptomatic complaints, may not really be normal women in strict sense, thus they may be having latent or an occult or commensal papillomavirus infection, that is, the presence of HPV DNA in the absence of a visible, histologic or cytologic abnormality. Oriel (1971) coined the lag period between exposure to HPV infection and development of clinical disease as ‘latent infection’, which was approximately 4 months, which is corroborated by Kreider *et al* (1987) experimentally, in their studies. The reason may be that the virus might be present in latent state and/or in unintegrated form without affecting cellular morphology, which shows up in Pap test. This transient infection may be cleared by immune system in majority of women in due course of time. However, those persisting may be integrated into the host cell genome and progress to carcinoma. It is also known that transformation of HPV appears to involve HPV DNA integration into host genome. Further the progress and outcome of an HPV infection depend on the HPV type, viral load and the nature and timing of local and tissue influences (Cheng *et al*, 1995; Jian *et al*, 1998).

HPV type 16 was found to be the most prevalent type (84.7%) which is in agreement with other studies, while the frequency of HPV type 18 (3.38) is very low when compared to other ethnic population (Durst *et al*, 1983; Yoshikawa *et al*, 1985; Das *et al*, 1992b; Bosch *et al*, 1995; Clifford *et al*, 2003) but it is consistent with Indian data (Das *et al*, 1992a, 1992b). Absence of any HPV type in seven cervical tumours indicates that some other mechanism/factors or a hitherto unknown HPV-dependent pathway also exists for the genesis of cervical cancer. The overall HPV positivity in 55 cervical dysplastic lesions including carcinoma-*in-situ* was 69.1%. The results were comparable to earlier published reports (van Den Brule *et al*, 1991; Cornelissen *et al*, 1992; Cuzick *et al*, 1994; Holowaty *et al* 1999; Voglino *et al*, 2000; Reddy *et al*, 2001). The positivity of high risk HPVs 16 and 18 was found to be 64.5%. One CIS sample was positive for both HPV 16 and HPV 18. The break up of HR-HPV type 16 positivity in mild, moderate, severe and in CIS was 54.5, 55.5, 75.0 and 83.3%, respectively. Similar prevalence was shown for HPV type 16 and 18 in dysplastic lesions by other authors (van Den Brule *et al*, 1991; Cornelissen *et al*, 1992; Cuzick *et al*, 1994; Holowaty *et al*, 1999; Voglino *et al*, 2000). Observation of a higher frequency of HR-HPV types in women with severe dysplastic lesions and CIS than in mild dysplsia is also in agreement with earlier reports (McCance *et al*, 1985; Cornelissen *et al*, 1992; Cuzick *et al*, 1994; Delvenne *et al* 1994). Gradual increase in the frequency of high risk HPV types 16 and 18 from mild to moderate to severe dysplastic lesions to invasive cervical cancer suggests that the frequency of high risk HR-HPV infection changes as a function of severity of cervical lesions.

Besides being an important risk factor for cervical cancer, HPV has been found to activate telomerase with its E6 oncoprotein (Klingelhutz *et al*, 1996). *Ex vivo* studies showed that transfection of normal epithelial cervical keratinocytes with the HPV E6 gene resulted in telomerase activation even before the occurrence of ‘crisis’ (Klingelhutz *et al*, 1996). It has been observed that low-grade dysplasias with the infection of HR-HPV 16 and 18 showed a higher rate of progression to malignancy (Reid *et al*, 1987; Das *et al*, 1989; Cuzick *et al*, 1992). It suggests that those early lesions infected with high risk HPV 16 could be induced to progress because of induction of telomerase activity by their E6 oncoprotein.

Activation of telomerase at a site, which is normally telomerase negative, indicates the presence of immortal or malignant cells (Hiyama *et al*, 1995; Sommerfield *et al*, 1996). Reddy *et al* (2001) reported 100% telomerase positivity in 29 cases of cervical intraepithelial neoplasia (CIN) IB, IIB, IIIA and IIIB. Several authors (Kyo *et al*, 1997; Meeker and Coffey, 1997; Zheng *et al*, 1997; Shroyer *et al*, 1998; Wisman *et al*, 2000) in survey on human malignancies reported 88–100% telomerase activity. Cervical cancers exhibit high telomerase activity irrespective of histopathology grading but the intensity/level of telomerase activity increased with the clinical progression of the disease.

In the present study, we observed weak telomerase activity in seven (64.6%) of 11 mild dysplasia samples. All 18 moderate, 26 severe dysplasias which included six CIS samples were positive for moderate to high to very high telomerase activity, respectively. High telomerase activity in all samples of CIS, at par with invasive carcinomas, is justified since this stage is a pre-invasive stage. Similar results were reported by several other authors (Kyo *et al*, 1997; Zheng *et al*, 1997; Shroyer *et al*, 1998; Zhang *et al*, 1999; Wisman *et al*, 2000). Meeker and Coffey (1997) noted 46% telomerase positivity in cervical precancerous tissues where as Snijders *et al* (1998) reported low telomerase positivity in CIN lesions. The high percentage of telomerase activity in the present study group may be due to the presence of more number of severe dysplasia cases. The very presence of telomerase activity in the preneoplastic cervical tissues indicates that telomerase is activated early in the course of cervical carcinogenesis and may be a vital constituent of malignant progression. The HR-HPV 16/18 infection showed 88.1% sensitivity in invasive carcinomas compared to 100% sensitivity of telomerase activity. It is further observed that as the positivity of oncogenic HPV types 16/18 increased telomerase activity also increased with the increased severity of the disease. Severe dysplasia cases showed 100% sensitivity for telomerase activity compared to 76.9% for HPV. This indicates a good association between HPV infection and activation of telomerase during cervical carcinogenesis.

Cervical scrapes collected from the same cervical cancer patients (*n*=59) also revealed similar results suggesting that the telomerase activity of exfoliated cells reflects that of the lesions or tumour tissues. This is a very interesting finding which indicates that cervical exfoliated cells can be used for the detection of telomerase activity. It will help in easy screening of women for telomerase activity along with Pap test in the same scraped cervical cells. Possible inhibition of the TRAP assay by the presence of normal cervical cells is not going to interfere with sensitivity of the assay since Kyo *et al* (1997) observed that the detection limit of TRAP assay was 100 cancer cells. Furthermore, Wisman *et al* (2001) successfully assayed telomerase activity even in 10 cervical cells.

We observed a positive association between telomerase activity and infection with HR-HPV type 16/18. These findings are in accordance with earlier reports (Zheng *et al*, 1997), which suggest telomerase activation as an early event occurring during cervical neoplastic transformation and HPV infection (Yashima *et al*, 1998). Consequently, monitoring telomerase activity could also have potential prognostic significance.

When we assessed association of HR-HPV types 16 and 18 with telomerase activity, we observed that, of seven samples of mild dysplasia that were positive for telomerase activity, six of these were positive for HR-HPV type 16 and one for low risk HPV type 11. Absence of telomerase activity in four mild dysplasia samples suggests that they belong to those groups of cases who may not progress since there are some percentages of dysplastic lesions, which revert back to normalcy. During the study of biology of low-grade squamous intraepithelial lesions (LSILs) using PCR based clonality assay, Park *et al* (1996) revealed that LSILs include two types of lesions that are biologically distinct; one is monoclonal and associated with malignant HPV types while the other is polyclonal and associated with other HPV types. As monoclonality is a hallmark of neoplasia irrespective of organ type, the lesions that express telomerase activity appears to have the characteristics of neoplastic lesions. As reactivation of telomerase activity is linked with malignancy, a follow-up of these samples only can determine the fate of these samples. Weak telomerase activity in seven normal cervical scrapes which were positive for HR-HPV types 16 and 18 makes an interesting observation. The cytology reports for these samples were inflammation (*n*=2) and ASC-US (*n*=1) and the colposcopic examination revealed cervical erosion in four cases. This is in agreement with earlier studies that showed presence of mild telomerase activity even in ASC-US, inflammation and cervical erosions (Kyo *et al*, 1997; Shroyer *et al*, 1998; Wisman *et al*, 2000). It is also known that the presence of HPV 16 E6 protein can activate telomerase activity irrespective of cells entering ‘crisis’ stage (Klingelhutz *et al*, 1996).

It has been established that there is variation in the interpretation of ASC-US Pap smears even among expert cytopathologists (Sherman *et al*, 1994). ASC-US/LSIL Triage Study (ALTS) recommends HPV DNA testing in women with ASC-US but not in LSIL (Sherman *et al*, 2002). This brings forth the debate over the subject that whether all women with abnormal cervical smear should be referred and treated (Flannelly and Kitchener, 1995). It may look too radical since only 1% of women with CIN I are estimated to progress to invasive cervical cancer (Holowaty *et al*, 1999). In addition, identifying women with abnormal cervical changes put such women under stress that they are at a risk of developing cervical cancer, when in fact majority of them never develop the disease (Raffle *et al*, 1995). The presence of telomerase activity in the whole range of cervical samples from mild dysplasia to stage IV cancers suggests that telomerase is activated early and play an essential role during cervical carcinogenesis. The average telomerase activity increased with the progression of the clinical stage. The gradual elevation of telomerase activity from mild to moderate to high to very high telomerase activity in different dysplsia and invasive cancer samples indicate that with the progression of the disease there is a concomitant increase in the telomerase activity (Kyo *et al*, 1997; Shroyer *et al*, 1998: Wisman *et al*, 2001; Baege *et al*, 2002).

The levels of hTERT mRNA expression complemented well with the telomerase activity levels observed in different grades of cervical lesions to invasive cervical cancer samples. One mild dysplasia sample, which was negative for telomerase activity, expressed hTERT. This may be explained due to the upregulation of hTERT before the reactivation of telomerase activity. Similar observations were made in other cancers including cervical cancer by several authors (Kyo *et al*, 1997; Nakano *et al*, 1998; Shroyer *et al*, 1998; Wisman *et al*, 2001).

It is remarkable that high rates of cervical cancer deaths still occur despite the fact that cervical cancer is an excellent model for early detection due to long interval between the time of infection or initial lesions and development of invasive cancer with a well-known natural history. Early identification and intervention should have a significant impact on the reduction of cervical cancer morbidity and mortality. Identification of women at risk for cervical cancer would not only minimise the unnecessary follow-up visits a woman has to undergo, but would also avoid invasive procedures without compromising on the disease detection. We suggest that telomerase activation is a relatively early-stage event in cervical carcinogenesis, and this activation is associated with the initiation and progression of cervical lesions. Detection of telomerase activity may serve as a tool for reliable diagnosis and prognosis of cervical neoplasias along with cytology and HPV testing.

## Figures and Tables

**Table 1:** Telomerase activity in relation to HPV status in invasive, dysplastic and normal cervical lesions

**Type of cervical lesion**	**HR-HPV 16/18**			**Telomerase activity**		
	**Positve**	**Negative**	**OR (*P*-value)**	**Positive**	**Negative**	**OR (*P*-value)**
**(a)**	**(b)**	**(c)**	**(d)**	**(e)**	**(f)**	**(g)**
Invasive cervical carcinomas (*n*=59)	52 (88.1%)	7 (11.9%)	141.1 (<0.001)	59 (100.0%)	0	—a (<0.001)
Severe (Severe dysplasia and CIS) (*n*=26)	20 (76.9)%	6 (23.1%)	63.3 (<0.001)	26 (100%)	0	—a (<0.001)
Moderate (*n*=18)	10 (55.5%)	8 (44.5%)	23.8 (<0.001)	18 (100%)	0	—a (<0.001)
Mild dysplasia (*n*=11)	6 (54.5%)	5 (45.5%)	22.8 (<0.001)	7 (63.6%)	4 (36.4%)	32.3 (<0.001)
Normal cervical tissues (*n*=40)	2 (5.0%)	38 (95.0%)	—	2 (5%)	38 (95.0%)	— (<0.001)

HPV=human papillomaviruses; HR-HPV**=**high risk HPV; OR=odds ratio.

In column (b) and (e) percentages for abnormal lesions indicate sensitivities.

In column (c) and (f) percentages for normal lesion indicate specificity.

a OR could not be calculated due to 0 cell value.

**Table 2:** Association between telomerase activity and HPV status in invasive, dysplastic and normal cervical lesions

	**Telomerase activity**								
	**Invasive carcinomas (*n*=59)**			**Dysplasias (*n*=55)**			**Normal (40)**		
**HPV status**	**Present**	**Absent**	**Total**	**Present**	**Absent**	**Total**	**Present**	**Absent**	**Total**
Positive	52 (100%)	0 (0%)	52	34 (94.4%)	2 (5.6%)	36	2 (100%)	0 (0%)	2
Negative	7 (100%)	0 (0%)	7	15 (78.9%)	4 (21.1%)	19	0 (0%)	38 (100%)	38
Total	59	0	59	49 (89.1%)	6 (10.9%)	55	2	38	40
									
% Agreement between HPV and telomerase activity	88.1			69.1			100		

HPV=human papillomaviruses.

Figures in parenthesis indicate percentages out of HPV status.
